# Cannabinoid Receptor Modulation of Neurogenesis: ST14A Striatal Neural Progenitor Cells as a Simplified In Vitro Model

**DOI:** 10.3390/molecules26051448

**Published:** 2021-03-07

**Authors:** Erika Cottone, Valentina Pomatto, Stefania Rapelli, Rosaria Scandiffio, Ken Mackie, Patrizia Bovolin

**Affiliations:** 1 Department of Life Sciences and Systems Biology, University of Turin, 10123 Torino, Italy; erika.cottone@unito.it (E.C.); valentina.pomatto@gmail.com (V.P.); stefania.rapelli@unito.it (S.R.); rosaria.scandiffio@unito.it (R.S.); 2 Department of Psychological and Brain Sciences, Gill Center for Biomolecular Science, Indiana University, Bloomington, IN 47405, USA; kmackie@indiana.edu

**Keywords:** endocannabinoid system, cannabinoid receptor, CB1, CB2, CB ligands, antagonists, neural progenitors, ST14A, proliferation, neurogenesis

## Abstract

The endocannabinoid system (ECS) is involved in the modulation of several basic biological processes, having widespread roles in neurodevelopment, neuromodulation, immune response, energy homeostasis and reproduction. In the adult central nervous system (CNS) the ECS mainly modulates neurotransmitter release, however, a substantial body of evidence has revealed a central role in regulating neurogenesis in developing and adult CNS, also under pathological conditions. Due to the complexity of investigating ECS functions in neural progenitors in vivo, we tested the suitability of the ST14A striatal neural progenitor cell line as a simplified in vitro model to dissect the role and the mechanisms of ECS-regulated neurogenesis, as well as to perform ECS-targeted pharmacological approaches. We report that ST14A cells express various ECS components, supporting the presence of an active ECS. While CB1 and CB2 receptor blockade did not affect ST14A cell number, exogenous administration of the endocannabinoid 2-AG and the synthetic CB2 agonist JWH133 increased ST14A cell proliferation. Phospholipase C (PLC), but not PI3K pharmacological blockade negatively modulated CB2-induced ST14A cell proliferation, suggesting that a PLC pathway is involved in the steps downstream to CB2 activation. On the basis of our results, we propose ST14A neural progenitor cells as a useful in vitro model for studying ECS modulation of neurogenesis, also in prospective in vivo pharmacological studies.

## 1. Introduction

The endocannabinoid system (ECS) is comprised of several different components: (a) the cannabinoid receptors, the best characterized being CB1 and CB2 receptors; (b) the endogenous cannabinoids, also called endocannabinoids (eCBs), among which anandamide (AEA) and 2-arachidonoylglycerol (2-AG); (c) the enzymes involved in eCB biosynthesis “on demand”, e.g., *N*-acylphosphatidylethanolamine-specific phospholipase D-like hydrolase (NAPE-PLD) and diacylglycerol lipase (DAGL); (d) the enzymes involved in eCB degradation, e.g., fatty acid amide hydrolase (FAAH) and monoacylglycerol lipase (MAGL); (e) the molecules involved in eCB transport across the membrane [1,2,3]. Additionally, various natural exogenous cannabinoids do exist, the most potent of which is Δ^9^-tetrahydrocannabinol (THC), the main psychoactive component of *Cannabis sativa* [4]. Considering that marijuana is one of the most abused substances in the world and it is becoming legal in many countries, a particular concern is on the fact that acute and chronic use of cannabis could lead to cognitive impairments; interestingly, not only chronic treatment with THC, but also the administration of a single ultra-low dose of THC was shown to lead to long-term cognitive impairments, possibly resulting from deficits in attention or motivation [5]. A noteworthy finding is the fact that THC induces striatal dopamine release in animals and humans [6], thus explaining some of the adverse effects of cannabis consumption on neuropsychiatric disorders, such as schizophrenia, and also suggesting that THC could share addictive properties with other drugs of abuse.

The ECS shares mediators and overlaps with other metabolic processes, thus recently an “expanded endocannabinoid system” or “endocannabinoidome” has been defined [7].

CB1 and CB2 cannabinoid receptors are seven-transmembrane G protein-coupled receptors [8]; they primarily signal through G_i/o_ proteins, leading to the inhibition of adenylyl cyclase and activation of Mitogen-activated protein kinases (MAPKs). In response to CB1 stimulation, MAPKs such as ERK1/2, c-Jun N-terminal kinase (JNK) and p38 are activated; CB1 was also shown to activate the Phosphoinositide 3-kinases (PI3K) pathway, thus leading to the regulation of neuronal survival. Similar to the CB1 receptor, the stimulation of CB2 has been demonstrated to promote neuronal survival through the activation of the PI3K/AKT/mTORC and JNK pathway; also, a Phospholipase C (PLC)-mediated intracellular calcium increase has been shown to activate MAPKs. Apart from these canonical signaling pathways, cannabinoid receptors are also able to signal through other non-canonical ways, such as activation of G_s_ and G_q_ proteins; also, complex crosstalk among cannabinoid receptors and other receptors, leading to heterodimerization and transactivation, has been shown [8]. Interestingly, different ligands can elicit different signaling pathways mediated by cannabinoid receptors [1].

CB1 is the most abundant G-protein coupled receptor in the mammalian brain; it is highly expressed by neurons in the cortex, amygdala, hippocampus, basal ganglia, and cerebellum, its activation leading to the modulation of neurotransmitter release [9]. The CB1 cannabinoid receptor has a pivotal role in neuroprotection, control of excitotoxicity, the survival of neural cells, as well as proliferation, differentiation and migration processes of neural progenitors (NPs) [10,11,12].

Different from CB1, the CB2 receptor is mostly distributed peripherally, especially in cells of the immune system [13], with a prevalent immunomodulatory role. However, recent studies showed CB2 expression also in the central nervous system [14], especially in association with neurodegenerative disorders [15]. In the adult brain, the CB2 receptor is localized in microglia, brain stem neurons, striatal neurons, hippocampal glutamatergic neurons, and dopaminergic neurons of the ventral tegmental area; CB2 mRNA levels are 100–200 times less abundant than CB1 mRNA, being however strongly upregulated in response to chronic pain, neuroinflammation and stroke [11]. Interestingly, the CB2 receptor is expressed in oligodendrocyte progenitors and neural progenitors [16,17,18] and it has been demonstrated that its activity is important in the control of adult neurogenesis under pathological conditions [12]. Indeed, the involvement of the CB2 receptor in neurodegenerative and neuroinflammatory disorders stimulated research toward CB2-targeted pharmacological approaches [2]. A substantial body of evidence suggests that the ECS modulates the proliferation, migration, specification and survival of neural progenitors in the developing and adult CNS [10]. During the different phases of neurogenesis in pre- and post-natal brain, all the ECS components are differentially expressed, e.g., 2-AG is prevalent in the prenatal period and dramatically decreases postnatally, while anandamide levels increase postnatally [19]. Interestingly, NPs commonly co-express CB1 and CB2 receptors; upon commitment to a neuronal fate, CB1 levels become up-regulated at the expense of CB2. CB2 seems more linked to a precursor undifferentiated proliferative state [16,17,18,20,21] and its involvement in axon guidance along the forming retino-thalamic pathway in vitro and in vivo has also been demonstrated [22]. Studies showed that 2-AG can act both on CB1 and CB2 receptors present in NPs derived from the subventricular zone, thus regulating cell proliferation and affecting neuroblast migration towards the olfactory bulbs [23,24,25].

Due to the complexity of the ECS, the full understanding of how its various components may contribute to control neurogenesis in developing and the adult brain is difficult to reach by in vivo approaches. A simplified in vitro model of neural progenitor cells could therefore be a useful tool to better understand the role of ECS components and to identify the intracellular mechanisms involved, as well as to provide the basis for ECS-targeted pharmacological approaches.

In this paper, we used the ST14A cell line, immortalized neural precursor-derived primary cells, dissociated from the rat striatal primordia at embryonic day 14 and conditionally immortalized by retroviral transduction of the temperature-sensitive variant of the SV40 large T antigen [26]. At the permissive conditions of 33 °C and 10% serum-containing medium, ST14A cells show high proliferative activity, while switching to the non-permissive temperature of 39 °C or at low serum concentrations [26,27,28] the cells stop or slow down their proliferation and start differentiating into striatal medium-sized spiny neurons [26,28,29]. Due to their simplicity of in vitro culturing, the possibility to be easily transfected and to be transplanted into an adult and developing rodent brain, ST14A has been successfully used by many authors to investigate several processes correlated to neural progenitors development and migration [30,31,32,33], as well as a model for studying Huntington’s disease [34,35].

In our research, we tested the suitability of ST14A cells as a simplified in vitro model for studying ECS modulation of neurogenesis. First of all, we assessed the expression of the ECS components necessary to a functional endocannabinoid system. Then, by using CB1/CB2 agonists and antagonists, we evaluated the effects of CB1 and CB2 receptor modulation on neural progenitor proliferation. Finally, we began to characterize the intracellular pathways involved in the CB2-regulated proliferation of striatal projection neuron progenitors.

## 2. Results

### 2.1. The Endocannabinoid System Is Expressed in ST14A Striatal Neural Progenitor Cells

**Figure 1:**
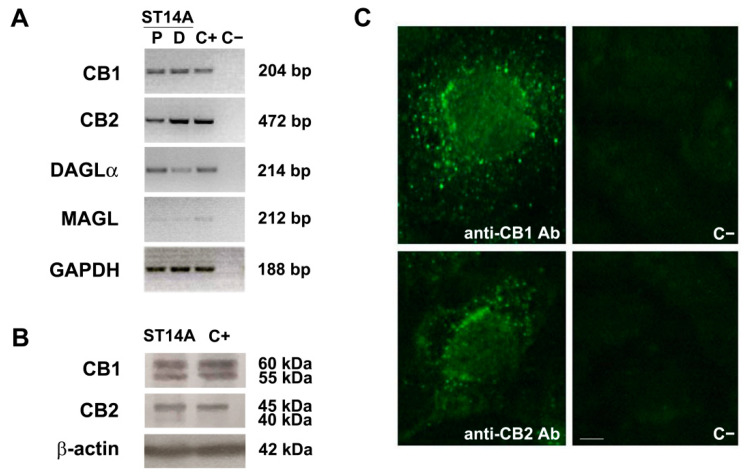
Endocannabinoid system expression in ST14A neural progenitor cells. (**A**) RT-PCR revealed specific bands corresponding to CB1 and CB2 receptors and to the enzymes DAGLα and MAGL in ST14A cells cultured 48 h under proliferating conditions (P). The same genes were also expressed in ST14A cells induced to differentiate toward a neuronal phenotype for 72 h (D, differentiating conditions). The housekeeping gene GAPDH was used as reference gene. The base pair (bp) length of the different amplicons is indicated. C+: positive controls, i.e., cDNA from rat brain or from rat spleen (for CB2 only); C−: negative control (no RT). (**B**) Western blot showing the expression of CB1 and CB2 receptors in ST14A neural progenitors (cells cultured 48 h under proliferating conditions); β-actin protein expression was used as a quality control of the protein extract. The apparent molecular weights of the bands are indicated (kDa). C+: positive controls, i.e., protein extracts from rat brain or spleen (for CB2 only). (**C**) Immunofluorescence for CB1 and CB2 receptors in ST14A cells neural progenitors (cells cultured 48 h under proliferating conditions). Single-cell magnification. Specific immunoreactivities are mainly distributed in the cytoplasm and in proximity to the nucleus. C−: negative controls, i.e., anti-CB1 or anti-CB2 pre-adsorbed antibodies. Calibration bar: 5 µm.

The expression of ECS components was evaluated in the ST14A striatal neural progenitor cell line. Cells were cultured for 48 h under permissive, proliferating conditions (P; 10% serum-containing medium, incubation at 33 °C). By means of qualitative RT-PCR, ST14A cells were shown to express mRNAs encoding for CB1 and CB2 receptors; moreover, diacylglycerol lipase α (DAGLα) and monoacylglycerol lipase (MAGL), mainly involved respectively in the biosynthesis and degradation of 2-AG, were also expressed (Figure 1A). Focusing on cannabinoid receptors, CB1 and CB2 expression was also investigated at the protein level by Western blot analysis (Figure 1B). In the case of CB1, two bands with apparent molecular weights around 60 and 55 kDa, possibly corresponding to differently glycosylated forms, were observed. The Western blot for CB2 receptor revealed instead a major band of 45 kDa and a weaker band of about 40 kDa. CB receptor cellular localization was then assessed by means of immunofluorescence (Figure 1C); CB1 and CB2 immunoreactivities were abundantly found in the cytoplasm, especially around the nucleus.

The expression of the ECS was also evaluated in ST14A cells cultured for 72 h in differentiating conditions (D; 0.5% serum-containing medium, incubation at 33 °C), which favors a reduction of cell proliferation and allows the progenitors to start the differentiation toward striatal medium-sized spiny neurons. Differentiating ST14A cells were found to express the mRNAs encoding for both CB1 and CB2 receptors, as well as for DAGLα and MAGL (Figure 1A).

The detection of cannabinoid receptors mRNAs and proteins, as well as the expression of the mRNAs encoding endocannabinoid synthetic and degradative enzymes, strongly support the presence of an active ECS in ST14A neural progenitor cells.

### 2.2. The Pharmacological Blockade of Cannabinoid Receptors Does Not Affect ST14A Cell Number

In order to determine if ST14A progenitor proliferation is under constitutive endocannabinoid regulation, we tested the effects of CB1 and CB2 pharmacological blockade.

**Figure 2:**
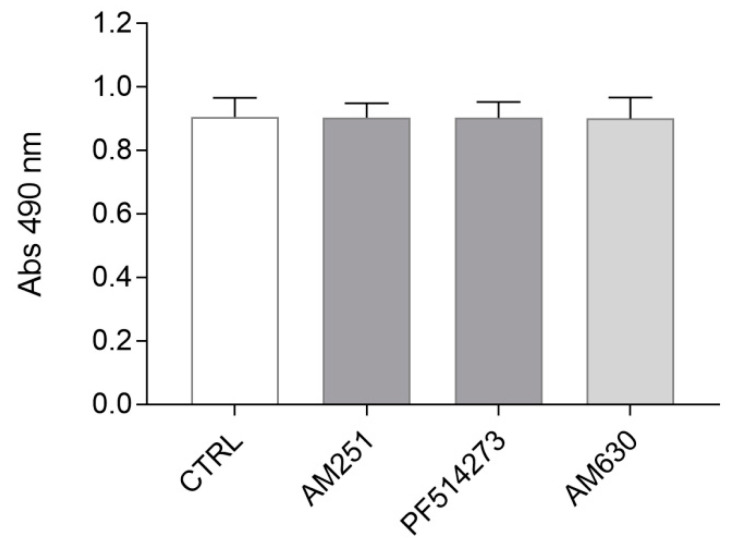
Evaluation of CB1/CB2 blockade effects on ST14A cell number. ST14A were treated for 24 h with 250 nM AM251 or 50 nM PF514273 (CB1 antagonists), or 300 nM AM630 (CB2 antagonist), then an MTS assay was performed. No effect on ST14A cell number was observed in treated cells, in respect to control (CTRL, medium plus 0.05% DMSO). Data from the MTS assay are expressed as mean ± standard deviation (SD) of the absorbance (λ = 490 nm); n = 8 replicates, 3 independent experiments.

Cells were cultured in proliferating conditions (at 33 °C, in 10% serum-containing medium) and treated for 24 h with alternatively one of the two selective CB1 antagonists AM251 and PF514273, and the CB2 antagonist AM630. A dose–response experiment (Figure S1) was conducted in order to verify different antagonist concentration effects on cell number, as well as to exclude cytotoxic effects and to select the best antagonist concentration to be used in subsequent experiments; based on previously unpublished experiments performed in our lab, a 24 h treatment was chosen. As shown in Figure 2 and Figure S1, neither CB1 nor CB2 blockade, at any antagonist concentration used, led to a change in ST14A cell number compared to untreated control cells, suggesting that constitutive cannabinoid signaling is not involved in ST14A cell proliferation/survival.

### 2.3. Exogenous Administration of the Endocannabinoid 2-AG and the CB2 Agonist JWH133 Induces ST14A Cell Proliferation through CB2 Receptor Activation

We subsequently tested whether exogenous activation of CB1 and CB2 receptors by the administration of the CB1/CB2 agonist 2-AG could affect ST14A cell proliferation. Cells were stimulated for 24 h under proliferating conditions with 2-AG, then an MTS assay was performed. A preliminary dose–response experiment (Figure S2, panel A) allowed us to select the optimal agonist concentration to be used in this and subsequent experiments; based on previously unpublished experiments performed in our lab, a 24 h treatment was chosen.

**Figure 3:**
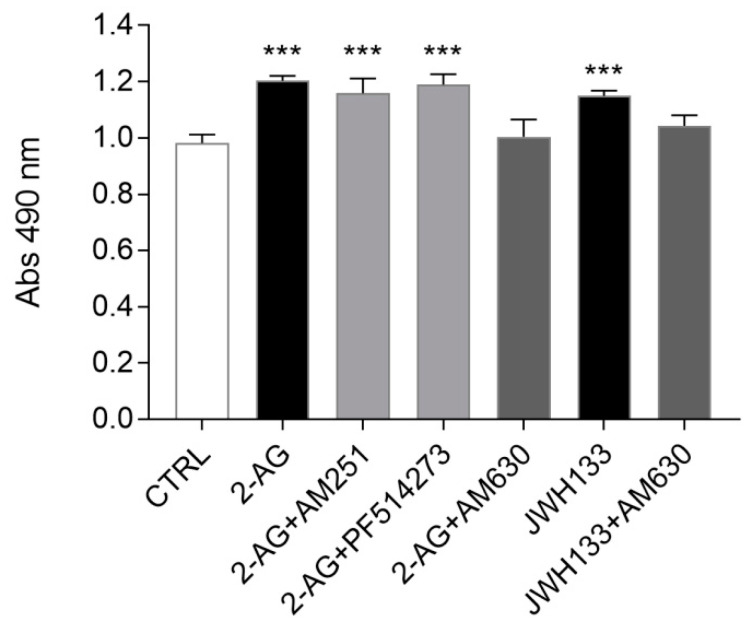
Evaluation of the effects of 2-AG and JWH133 alone or in the presence of specific CB1/CB2 antagonists on ST14A cell number. 2-AG (5 μM) induces an increase of ST14A cell number after 24 h, with respect to control cells (CTRL). The effect was not reverted by the coadministration of 2-AG with the CB1 selective antagonists AM251 (250 nM) and PF514273 (50 nM); on the other hand, cell number was comparable to control when 2-AG was coadministered with the selective CB2 antagonist AM630 (300 nM). Similarly, the selective CB2 receptor agonist JWH133 (300 nM) induced ST14A cell number increase, which was specifically reverted by the coadministration of AM630 (300 nM). Data from the MTS assay are expressed as means ± SD of the absorbance (λ = 490 nm); n = 5 replicates, 3 independent experiments. *** = *p* ≤ 0.001 vs. control.

As shown in Figure 3, 2-AG (5 μM) was able to induce a statistically significant increase in ST14A cell number, compared to control levels. To clarify the receptor subtype involved, CB1 and CB2 selective antagonists, used at the previously selected concentrations, were coadministered with 2-AG. 2-AG (5 μM) effects were not modified by co-treatment with the CB1 antagonists (250 nM AM251 or 50 nM PF514273), thus excluding a CB1-mediated effect. On the other hand, the 2-AG-mediated increase in ST14A cell number was specifically reverted by the coadministration of the CB2 selective antagonists AM630 (300 nM), indicating the involvement of CB2 receptor. This result was further confirmed by treatment for 24 h with a selective CB2 receptor agonist; indeed, JWH133 (300 nM; see Figure S2, panel B for preliminary dose–response experiment) induced an increase in cell number, in respect to control, and the effect was specifically reverted by coadministration of AM630 (300 nM) (Figure 3).

**Figure 4:**
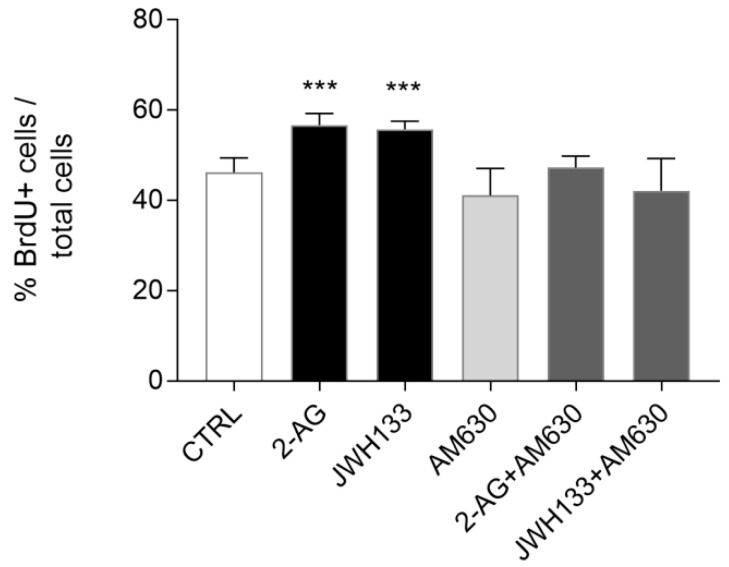
Evaluation of the effects of 2-AG and JWH133 alone or in the presence of the specific CB2 antagonist AM630 on ST14A cell proliferation rate. Results are shown as ratio (%) of the number of BrdU+ cells over the number of total cells, stained with DAPI fluorophore. After 24 h treatment with 2-AG (5 μM) and JWH133 (300 nM) an increase in BrdU incorporation was observed, with respect to untreated cells (CTRL); the effect was specifically reverted in both the cases by 300 nM AM630 coadministration. Data are expressed as mean ± standard deviation (SD); n = 3 replicates (5 random fields counted in each well), 3 independent experiments. *** = *p* ≤ 0.001 vs. control.

To confirm that the increase in ST14A cell number observed after 2-AG and JWH133 treatment was the result of an increase in cell proliferation and not in the survival rate, a BrdU-based proliferation assay was performed. Both the endocannabinoid 2-AG (5 μM) and the synthetic CB2 agonist JWH133 (300 nM) significantly increased BrdU incorporation in ST14A cells, thus indicating a proliferative effect (Figure 4). AM630 (300 nM) administration alone did not influence ST14A proliferation rate, while its coadministration with 2-AG and JWH133 specifically blocked the proliferative effect induced by the agonists (Figure 4).

BrdU-based experiments demonstrated therefore that exogenously administered endocannabinoid 2-AG induces ST14A neural progenitor proliferation via a CB2-mediated mechanism.

### 2.4. PLC Pharmacological Blockade Impairs CB2-Mediated ST14A Cell Proliferation

In order to identify the possible intracellular effectors involved in CB2-mediated ST14A cell proliferation, we evaluated the effects of pharmacological blockade of the signalling cascades involving PLC and PI3K activation.

The day after seeding, ST14A cells were pre-incubated for 30 min with the PLC inhibitor U73122 (2 μM) or the PI3K inhibitor wortmannin (150 nM), then inhibitors were removed and cells were cultured for 24h in the presence of the agonists 2-AG or JWH133; cell counting was then performed with an MTS assay. The experiments were conducted in a medium containing 2% serum (instead of 10%) in order to reduce possible interference of serum components on the effects of PLC and PI3K inhibitors on cell proliferation.

**Figure 5:**
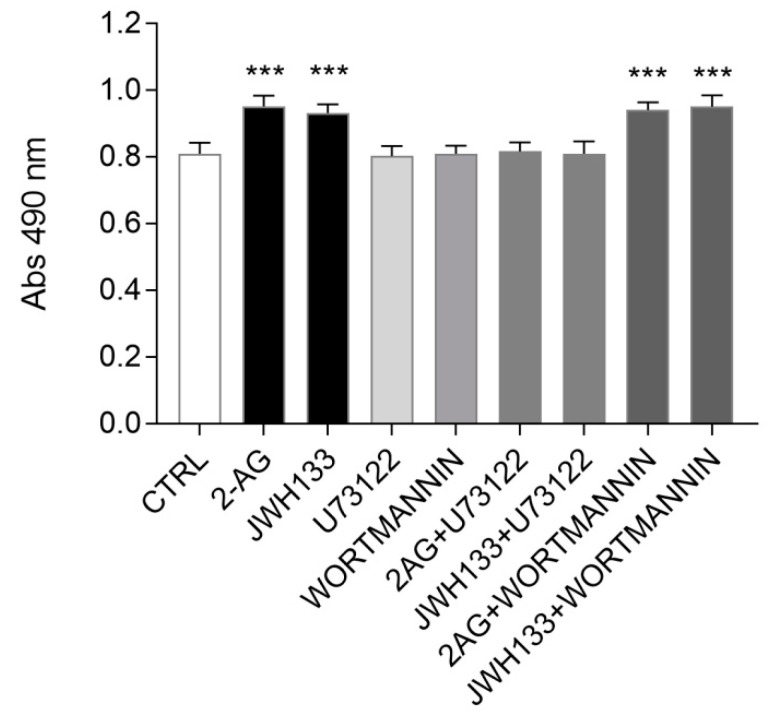
Evaluation of Phospholipase C (PLC) and PI3K pharmacological blockade on 2-AG- and JWH133-induced ST14A cell number increase. Cells were pretreated 30 min with U73122 (2 μM) or wortmannin (150 nM) prior to 2-AG (5 μM) or JWH133 (300 nM) incubation for 24 h in 2% serum-containing medium. U73122, but not wortmannin pretreatment, reverted 2-AG- and JWH133-driven ST14A cell number increase. Data from MTS assay are expressed as means ± standard deviation (SD) of the absorbance (λ = 490 nm); n = 8 replicates, 3 independent experiments. *** = *p* ≤ 0.001 vs. control.

As shown in Figure 5, at the concentrations used, the pre-treatment with the two inhibitors did not have any effect *per se* on ST14A viability/cell number, allowing further investigations. Interestingly, wortmannin pretreatment did not counteract the 2-AG- and JWH133-driven increase in cell number. On the opposite, the presence of the PLC inhibitor U73122 was able to revert the cell number increase induced by 2-AG- and JWH133 to control conditions.

Overall, our findings indicate that exogenously administered 2-AG promote ST14A neural progenitor proliferation through CB2 receptor engagement and PLC pathway activation.

## 3. Discussion

The endocannabinoid system modulates several biological processes, including the generation and survival of neurons in the developing and adult CNS, also under pathological conditions [10]. Indeed, the involvement of CB2 receptor in neurogenesis, as well as neurodegenerative and neuroinflammatory disorders, open to new possible pharmacological strategies based on the use of CB2-specific therapeutic drugs, possibly overcoming the neuropsychiatric adverse effects of CB1-targeted therapies [2,8].

In this work, we propose ST14A striatal neural progenitor cells as a simplified in vitro model suitable for studying the role of the ECS in neurogenesis, as well as for ECS-targeted pharmacological approaches.

The ST14A cell line was established by immortalization of neural precursor-derived primary cells dissociated from the rat striatal primordia at embryonic day 14 [26]. Compared to neuroblast primary cultures, this experimental model is easier to handle and to maintain in culture, so it is more suitable when approaching initial studies on molecular interactions. Furthermore, ST14A cells have also been used extensively for genetic manipulation experiments and transplantation into an adult and developing rodent brain, making these cells an in vitro system with great potential for biochemical, molecular, and biological studies correlated to neural progenitors development and migration [26,30,31,32,33], as well as a model for studying neurological diseases [34,35].

In this paper we showed that ST14A neural progenitor cells display an active ECS, in agreement with the findings obtained by Bari and colleagues [34] on ST TetOn 12.7, a ST14A subclone able to express reverse tetracycline-controlled transactivator under the control of doxycycline; also, previous studies on primary cultures of neural progenitors demonstrated functional CB1 and CB2 receptor expression [17,24,36,37]. In particular, we found that several components of the system, such as the cannabinoid receptors CB1 and CB2, as well as the endocannabinoid synthetic and degradative enzymes DAGLα and MAGL, are expressed in ST14A neural progenitors (cultured under permissive, proliferating conditions). Consistent with previous results [34], Western blot analysis confirmed the expression of both CB1 and CB2 receptors; CB1 receptor appeared as two bands with an apparent molecular weight around 60 and 55 kDa, possibly corresponding to differently glycosylated forms, while in the case of CB2 receptor a major band of 45 kDa and a weaker band of about 40 kDa were seen. In ST TetOn 12.7, instead, CB1 and CB2 receptors were detected as single bands of 60 kDa and 45 kDa, respectively [34]; the discrepancy with our results could be either due to the different ST14A clone and/or the different primary antibodies used. Immunofluorescence analysis for CB1 and CB2 revealed, accordingly with [34], cytoplasmic localizations of both the receptors, rather than a membrane localization, probably due to their intense trafficking and internalization; a marked perinuclear localization was found for CB2, according to the observations reported by [38]. Actually, CB2 binding sites were demonstrated to be predominantly located intracellularly in prefrontal cortical pyramidal neurons [39] and functional CB2 receptors were demonstrated at the endo-lysosome level [40]; also, CB1 receptor localization is not exclusively on the plasma membrane, since active CB1 were localized also in the outer membrane of mitochondria [41] and a predominant intracellular localization have been observed in diverse cell types and also undifferentiated neuronal cells [42]. In our study, we also widened our analysis to ECS expression in ST14A cells induced to differentiate toward a medium-size spiny neuron phenotype; mRNAs encoding for CB1 and CB2 receptors, as well as for DAGLα and MAGL were detected, consistent with studies showing the presence of a functional ECS in the striatum [14,43].

We subsequently focused our attention on the possible modulation played by the ECS on ST14A neural progenitor proliferation [44,45].

First, we assessed the effects of a perturbation of the endogenous ECS by pharmacological blockade of the cannabinoid receptors. Under these conditions, neither CB1 nor CB2 blockade had significant effects on ST14A neural progenitor cell number. On the other hand, the stimulation of cannabinoid receptors with a non-selective CB1/CB2 ligand, the endocannabinoid 2-AG, induced ST14A cell number increase through the activation of the CB2 receptor, as indicated by the fact that the effect was reverted by the coadministration of 2-AG with the CB2 specific antagonist AM630, but not with the CB1 antagonists AM251 and PF514273. CB2 involvement was further supported by the finding that the CB2 specific synthetic agonist JWH133 increased ST14A cell number, an effect reversed by the coadministration of the CB2 antagonist. Interestingly, a BrdU assay allowed us to demonstrate that the increase in ST14A cell number was related to an enhancement of neural precursor proliferation rate, rather than an increase in cell survival. In our study, we chose to use the endogenous ligand 2-AG to better mimic the ECS in an embryonic environment, since until birth 2-AG is much more abundant than AEA [19]. Our results about CB2-mediated proliferation are in agreement with the findings of [16,17,23,24], showing that endocannabinoids and synthetic cannabinoids can act on CB2 receptors present in NPs, regulating cell proliferation. The fact that we did not observe any effect on neural progenitor cell number following CB2 blockade, while CB2 agonist administration induced cell proliferation, could possibly be explained by the fact that only a few receptors might be activated in basal conditions; conversely, following exposure to exogenous ligands, CB2 receptors are massively activated and the effect of inhibition could be readily visible.

We aimed also to identify some of the possible intracellular effectors involved in CB2-mediated ST14A cell proliferation. CB1 and CB2 share several downstream signaling mechanisms in neural progenitors [21]; in particular, they are coupled to the activation of the ERK and the PI3K/Akt pathways. In cerebellar progenitor cells, CB1-induced cell proliferation was shown to be mediated by the PI3K/Akt/GSK3β and in cortical progenitors, CB1 drives mTORC1 signaling and cell proliferation. CB2 was shown to promote hippocampal neural progenitor proliferation through activation of the PI3K/Akt/mTORC axis [17,46]. In addition, previous studies demonstrated that 2-AG-mediated activation of CB2 leads to a PLC-IP_3_R dependent intracellular calcium increase and subsequent massive activation of MAPK/ERK cascade [46]. By preincubation with the PI3K inhibitor wortmannin or the PLC inhibitor U73122, we observed that the inhibition of PI3K had no consequence on CB2 ligand-mediated proliferation, while the PLC inhibitor U73122 significantly impaired the process. The dissimilarity between our data and Palazuelos and colleagues’ observations [17] could be possibly due to the different brain areas of cell origin (hippocampus vs. striatum). Furthermore, cells belonging to different brain areas and ages (embryonic and adult) could display diverse intracellular cascades related to their stage-specific enzymatic equipment. Interestingly, intracellularly located CB2 receptors were demonstrated to open IP_3_R-dependent Ca^2+^-activated Cl^-^ channels in prefrontal cortex pyramidal neurons [46].

In conclusion, our study indicates that ST14A cells express a functional endocannabinoid system that is actively involved in the regulation of neural progenitor proliferation. ST14A cells could therefore represent a useful, simplified in vitro model for studying ECS modulation of neurogenesis. Moreover, the model could be used to test new therapeutic drugs acting on the cannabinoid system, thus providing the basis for in vivo pharmacological studies.

## 4. Materials and Methods

### 4.1. Cell Culture

ST14A striatal neural progenitor cell line (kindly provided by Dr. Elena Cattaneo, University of Milan, Milan, Italy) was cultured on 100 mm Petri-dishes (BD Biosciences, San Jose, CA, USA) in Dulbecco’s modified Eagle’s medium (DMEM) supplemented with 100 units/mL penicillin, 0.1 mg/mL streptomycin, 1 mM sodium pyruvate, 2 mM L-glutamine (all supplied by Sigma-Aldrich, St. Louis, MO, USA), and 10% fetal bovine serum (FBS, GIBCO^®^, Gaithersburg, MD, USA) decomplemented at 56 °C for 30 min. Cells were grown as monolayers at 33 °C in a 5% CO_2_ incubator.

### 4.2. RNA Extraction and RT-PCR

Cells were seeded and let grow under permissive, proliferating conditions at 33 °C for 48 h in DMEM containing 10% FBS. Only for gene expression studies, cells were also grown under non-permissive, differentiating conditions at 33 °C for 72 h in DMEM containing 0.5% FBS. Total RNA extraction was performed using TRIZOL^®^ Reagent (Invitrogen, Carlsbad, CA, USA) following the manufacturer’s instruction. DNA contaminants were eliminated using TURBO DNA-free kit (Applied Biosystems, San Francisco, CA, USA). cDNA was synthesized from total RNA by using Multiscribe RT (Applied Biosystems, USA) and random nonamers, starting from 2 µg of total RNA for each sample. PCR (30 amplification cycles) was performed using 250 ng cDNAs. Negative controls (C−) were carried out replacing cDNA with an equal amount of total RNA (no RT); as positive controls (C+), cDNAs from rat brain or rat spleen (for CB2 amplification only) were used. The housekeeping gene GAPDH was used as reference gene. Specific primers (Table 1) were designed on the basis of rat sequences, using both Primer3 (http://frodo.wi.mit.edu/primer3/, accessed on 15 December 2020) and AnnHyb (http://www.bioinformatics.org/annhyb/, accessed on 15 December 2020) programs, paying attention to choose primers on different exons to avoid amplification of genomic DNA.

PCR reaction products were separated by 2% agarose gel electrophoresis in 1X TAE buffer. The correct length of the amplicons was confirmed by analysis with Gel Doc system and the software Quantity One (BioRad, Hercules, CA, USA), using Low DNA Mass Ladder (Invitrogen, USA) as molecular weight standards.

### 4.3. Western Blot

Cells were seeded and let grow under proliferating conditions at 33 °C for 48 h in DMEM containing 10% FBS, then total proteins were extracted by lysing cells in boiling Laemmli buffer (2.5% SDS, 0.125 M Tris-HCl, pH 6.8), followed by 3 min denaturation at 100 °C. Protein concentration was determined by BCA kit (Sigma-Aldrich, USA). As positive controls, rat brain or rat spleen (for CB2) total proteins were used. Protein extracts (20 µg/lane) were subjected to 10% SDS polyacrylamide gel electrophoresis (SDS-PAGE) and then blotted onto nitrocellulose membranes (BioRad, Hercules, CA, USA) according to the manufacturer’s instructions. After blocking with 5% powder milk in TBS-T buffer (20 mM Tris, 150 mM NaCl, 0.1% Tween20, pH 7.4), filters were probed with anti-CB1 C-terminus (last 15 aa of CB1 rat receptor) or anti-CB2 N-terminus (first 39 aa of CB2 rat receptor) primary polyclonal antibodies (diluted 1:800 in TBS-T containing 1% no-fat milk); both the antibodies were produced in Ken Mackie’s lab (Indiana University, Bloomington, IN, USA). Membranes were then washed in TBS-T and incubated with an anti-rabbit IgG HRP-conjugated secondary antibody (1:5000 dilution; Amersham Biosciences, Little Chalfont, UK). In order to check protein integrity, the expression of the housekeeping protein β-actin was revealed by means of anti-β-actin monoclonal primary antibody (diluted 1:10,000; Sigma-Aldrich, USA) and anti-mouse IgG HRP-conjugated secondary antibody (1:5000 dilution; Amersham Biosciences, USA). Specific bands were visualized by using enhanced chemiluminescence (ECL) detection system (Amersham Biosciences, USA). The apparent molecular weights of the stained proteins were determined by analysis with Gel Doc system and the software Quantity One, using prestained protein ladders (PageRuler Plus, Fermentas, Waltham, MA, USA) as reference.

### 4.4. Immunofluorescence

ST14A cells were seeded on poly-L-lysine-coated coverslips (3500 cells/cm^2^). After 48 h of growth in DMEM containing 10% FBS, at 33 °C (proliferating conditions), cell were first rinsed in PBS containing Ca^2+^ and Mg^2+^, and then in 0.05% sucrose-PBS. Cells were subsequently fixed with 4% PAF in 0.1 M phosphate buffer (PB), pH 7.4 for 10 min. After 4 washings in PBS, cells were incubated in blocking serum (PBS containing 5% BSA, 10% normal serum, 0.1% TritonX-100) at RT for 1 h. Cells were then incubated O/N at 4 °C with the anti-CB1 or anti-CB2 primary antibody, diluted 1:400 or 1:200, respectively, in 0.01 M PBS plus 10% normal goat serum. Controls were set up by incubating cells with primary antibodies pre-adsorbed O/N with the specific immunogens. Cells were washed in PBS and incubated for 1 h with anti-rabbit IgG AlexaFluor 488-conjugated secondary antibody (1:250 dilution; Invitrogen, USA), washed again and mounted with 1,4-diazabicyclo [2.2.2]-octane (DABCO; Sigma-Aldrich, USA). Image analysis was performed with a Nikon fluorescent microscope coupled with a computer-assisted image analysis system (Neurolucida software, MicroBrightField, Williston, VT, USA).

### 4.5. Cell Count Assays

Cells were seeded at a density of 1500 cells/well in 96-well plates, in 200 μL DMEM containing 10% FBS and incubated at 33 °C. The following day, the medium was replaced with DMEM plus 10% FBS, containing alternatively or in combination (depending on the experiment) 5 μM 2-AG, 300 nM JWH133, 250 nM AM251, 50 nM PF514273, 300 nM AM630 (all purchased from Tocris Bioscience, Bristol, UK); for controls, medium plus 0.05% DMSO was used. Then, 24 h later, 20 µL of MTS Cell Titer 96 solution (Promega, Madison, WI, USA) was added and plates were incubated at 37 °C for 4 h. The absorbance was measured in a Microplate Reader (Bio-Rad, USA) at a wavelength (λ) of 490 nm. At least 5 replicates for each condition were set up and the experiment was repeated three times.

For PLC- and PI3K-blockade experiments, cells were seeded as before, then the medium was replaced with DMEM plus 2% FBS, containing 2 μM U73122 (PLC inhibitor, Sigma-Aldrich, USA), 150 nM wortmannin (PI3K inhibitor, Sigma-Aldrich, USA) or 0.05% DMSO (vehicle only) for 30 min. Then, all the wells were washed with PBS and the medium was replaced with DMEM plus 2% FBS, containing alternatively 5 μM 2-AG or 300 nM JWH133. Then, 24 h later, MTS assay was performed and cell density was measured following the protocol reported above.

### 4.6. Cell Proliferation Assay

Cells were seeded on poly-L-lysine coated coverslips in 10% FBS-DMEM at a density of 3500 cells/cm^2^. The following day, the medium was replaced with 10% FBS-DMEM with or without 5 μM 2-AG, 300 nM JWH133, 300 nM AM630 and cells were cultured for another 24 h. Six hours before cell fixation, BrdU (10 μM) was added to the culture medium, then cells were fixed with 4% PAF in 0.1 M PB, pH 7.4, for 10 min and processed for BrdU-immunocytochemistry and DAPI staining. Briefly, cells were washed in PBS, then incubated at 37 °C with 2N HCl for 30 min and washed for 10 min with boric acid (0.1 M, pH = 8.5). Cells were washed and incubated in blocking serum (0.01 M PBS plus 10% normal serum) at RT for 1 h. Cells were incubated O/N at 4 °C with anti-BrdU mouse monoclonal antibody (Sigma-Aldrich, USA; dilution 1:3000 in 0.01 M PBS and 10% normal serum). Cells were washed and incubated for 1 h with anti-mouse IgG Cy3-conjugated secondary antibody (dilution 1:400; Jackson ImmunoResearch, West Grove, PA, USA). For nuclear staining, cells were labelled with 4′,6-diamidino-2-phenylindole (DAPI) for 10 min and mounted with DABCO. Cell counts and image analysis were performed with a Nikon fluorescent microscope coupled with a computer-assisted image analysis system (Neurolucida Software, MicroBrightField, USA). Five random fields were counted in each well and each treatment was done at least in triplicate; the experiment was repeated three times. The cell proliferation was determined for each sample as the ratio of the number of BrdU+ cells over the total cell number (cells stained with DAPI).

### 4.7. Statistical Analysis

All the data were analyzed using commercially available software (SPSS version 26 for Windows; SPSS Inc., Chicago, IL, USA). Statistical analysis was performed using one-way ANOVA followed by Tukey’s and Bonferroni’s post hoc tests. The level of significance was set at *p* < 0.05.

## Figures and Tables

**Table 1:** List of the primers used for PCR analysis.

	PCR Primers	Annealing T (°C)
GAPDH	Fw: 5′-TGGCATTGTGGAAGGGCTCATGAC-3 Rev: 5′-ATGCCAGTGAGCTTCCCGTTCAGC-3′	60
CB1	Fw: 5′-GGGTTACAGCCTCCTTCACA-3′ Rev: 5′-CAGATTGCAGCTTCTTGCAG-3′	55
CB2	Fw: 5′-GGAGTACATGATCTTGAGTGAT-3′ Rev: 5′-AGAACAGGGACTAGGACAAC-3′	50
DAGLα	Fw: 5′-GGCAAGACCCTGTAGAGCTG-3′ Rev: 5′-TAAAACAGGTGGCCCTCATC-3′	60
MAGL	Fw: 5′-TAGCAGCTGCAGAGAGACCA-3′ Rev: 5′-GATGAGTGGGTCGGAGTTGT-3′	60

## Data Availability

The data presented in this study are available on request from the corresponding author.

## Supplementary Materials

The following are available online, Figure S1. Evaluation of the effect of different concentrations of AM251, PF514273 and AM630 on ST14A cell number. Figure S2. Evaluation of the effect of different concentrations of 2-AG and JWH133 on ST14A cell number.

## Sample Availability

Anti-CB1 and anti-CB2 primary antibodies are available from Ken Mackie (Indiana University, Bloomington, IN, USA).
