# Supplementary material for: Cannabinoid Receptor Modulation of Neurogenesis: ST14A Striatal Neural Progenitor Cells as a Simplified In Vitro Model
Source: Molecules. 2021 Mar 7;26(5):1448. doi: 10.3390/molecules26051448 (PMC7962126; doi:10.3390/molecules26051448)
Supplement: Supplementary file 1 [file molecules-26-01448-s001.pdf]

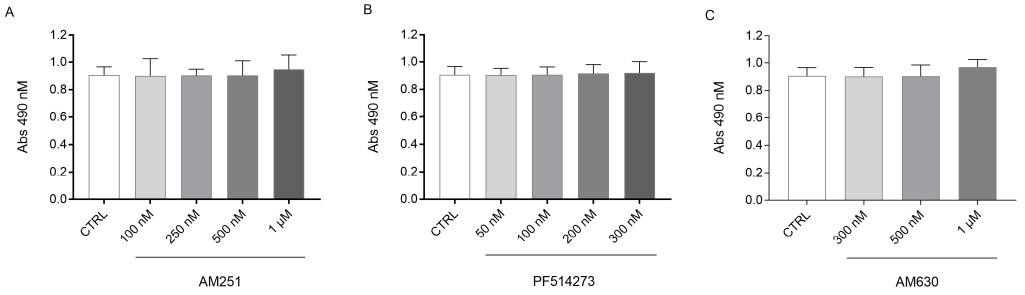

Figure S1. Evaluation of the effect of different concentrations of AM251, PF514273 and AM630 on ST14A cell number. A dose-response experiment was performed treating cells for 24h with **A)** 100 nM, 250 nM, 500 nM or 1 µM AM251 (CB1 antagonist); **B)** 50 nM, 100 nM, 200 nM or 300 nM PF514273 (CB1 antagonist); **C)** 300 nM, 500 nM or 1 µM AM630 (CB2 antagonist). None of the antagonists, at any concentration used, induced a statistically significant variation in ST14A cell number, in respect to control cells (CTRL), neither they had cytotoxic effects. AM251 (250 nM), PF514273 (50 nM) and AM630 (300 nM) were the antagonist concentrations chosen for subsequent experiments. Data from MTS assay are expressed as means ± standard deviation (SD) of the absorbance (λ=490 nm); n=8 replicates, 3 independent experiments.

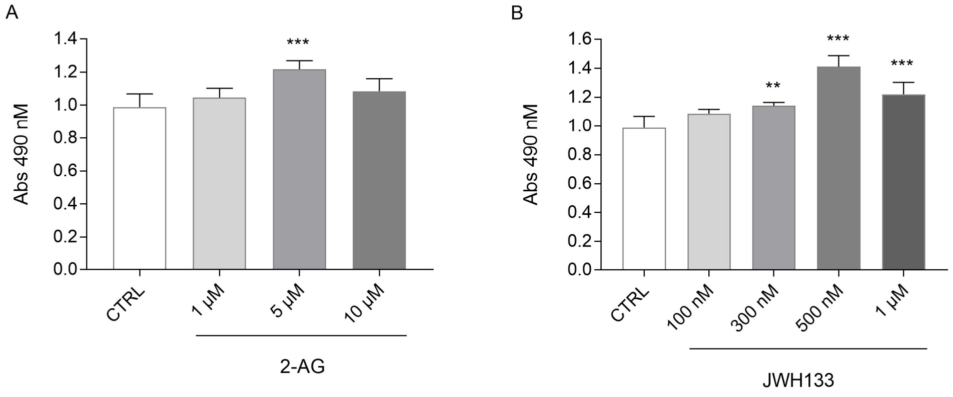

Figure S2. Evaluation of the effect of different concentrations of 2-AG and JWH133 on ST14A cell number. A preliminary dose-response experiment was performed treating cells for 24h with **A)** 1 µM, 5 µM or 10 µM 2-AG and **B)** 100 nM, 300 nM, 500 nM or 1 µM JWH133. 2-AG (5 µM), as well as the specific CB2 agonist JWH133 at all the concentrations tested, but 100 nM, induced ST14A cell number increase, in respect to control cells (CTRL). 2-AG (5 µM) and JWH133 (300 nM, the lower active dose) were the ligand concentrations chosen for subsequent experiments. Data from MTS assay are expressed as means ± standard deviation (SD) of the absorbance (λ=490 nm); n=5 replicates, 3 independent experiments. **=p≤ 0.01, ***=p≤ 0.001 vs. control.
